# Service evaluation of the implementation of a digitally-enabled care pathway for the recognition and management of acute kidney injury

**DOI:** 10.12688/f1000research.11637.2

**Published:** 2017-08-07

**Authors:** Alistair Connell, Hugh Montgomery, Stephen Morris, Claire Nightingale, Sarah Stanley, Mary Emerson, Gareth Jones, Omid Sadeghi-Alavijeh, Charles Merrick, Dominic King, Alan Karthikesalingam, Cian Hughes, Joseph Ledsam, Trevor Back, Geraint Rees, Rosalind Raine, Christopher Laing

**Affiliations:** 1 Centre for Human Health and Performance, University College London, 170 Tottenham Court Road, London, W1T 7HA, UK; 2 Institute of Sport, Exercise and Health, London, W1T 7HA, UK; 3 Department of Applied Health Research, University College London, 1-19 Torrington Place, London, WC1E 7HB, UK; 4 Population Health Research Institute, St George’s, University of London, Cranmer Terrace, London, SW17 0RE, UK; 5 Royal Free London NHS Foundation Trust, Pond Street, London, NW3 2QG, UK; 6 DeepMind Health, 5 New Street Square, London, EC4A 3TW, UK; 7 University College London, Gower Street, London, WC1E 6BT, UK

**Keywords:** nephrology, acute kidney injury, AKI, e-alert

## Abstract

Acute Kidney Injury (AKI), an abrupt deterioration in kidney function, is defined by changes in urine output or serum creatinine. AKI is common (affecting up to 20% of acute hospital admissions in the United Kingdom), associated with significant morbidity and mortality, and expensive (excess costs to the National Health Service in England alone may exceed £1 billion per year). NHS England has mandated the implementation of an automated algorithm to detect AKI based on changes in serum creatinine, and to alert clinicians. It is uncertain, however, whether ‘alerting’ alone improves care quality.

We have thus developed a digitally-enabled care pathway as a clinical service to inpatients in the Royal Free Hospital (RFH), a large London hospital. This pathway incorporates a mobile software application - the “Streams-AKI” app, developed by DeepMind Health - that applies the NHS AKI algorithm to routinely collected serum creatinine data in hospital inpatients. Streams-AKI alerts clinicians to potential AKI cases, furnishing them with a trend view of kidney function alongside other relevant data, in real-time, on a mobile device. A clinical response team comprising nephrologists and critical care nurses responds to these AKI alerts by reviewing individual patients and administering interventions according to existing clinical practice guidelines.

We propose a mixed methods service evaluation of the implementation of this care pathway. This evaluation will assess how the care pathway meets the health and care needs of service users (RFH inpatients), in terms of clinical outcome, processes of care, and NHS costs. It will also seek to assess acceptance of the pathway by members of the response team and wider hospital community. All analyses will be undertaken by the service evaluation team from UCL (Department of Applied Health Research) and St George’s, University of London (Population Health Research Institute).

## Introduction

Acute kidney injury (AKI) is a sudden loss of kidney function, defined by a rise in serum creatinine or a fall in urine volume ^1^. It has diverse causes, which include sepsis or acute infection, hypovolaemia or hypoperfusion, nephrotoxicity (from drugs or radiological contrast), obstruction of the renal tract, and primary renal diseases such as acute glomerulonephritis. In the United Kingdom, AKI affects up to 15% of hospital admissions and 20% of emergency admissions ^2, 3^. AKI may result in fluid overload, respiratory failure and metabolic derangements such as hyperkalaemia ^4^, and is thus strongly associated with adverse outcomes including death ^5^, prolonged hospitalisation ^6^, requirement for renal replacement therapy ^7^, and a need for high dependency or intensive care ^8^. It is also associated with an increased lifetime risk of chronic kidney disease ^9^. AKI is also expensive: the associated excess costs to the National Health Service (NHS) in England may exceed £1 billion per annum ^3^.

Management of AKI involves four processes of care: timely recognition, general supportive care, therapy directed at the underlying cause, and management of complications. Across the NHS, there are substantial cross-pathway deficits in care ^10^. Increasing awareness of these and of the clinical and economic impact of this condition has led to local, regional, national and global initiatives to try to prevent AKI from occurring, and to encourage timely and appropriate interventions to prevent progression and deliver more rapid recovery. Clinical practice guidelines for the management of AKI have now been developed ^11^.

More recently, on the basis that the prompt and reliable identification of AKI cases to clinicians may trigger improved care, NHS England issued a national patient safety alert on “standardising the early identification of Acute Kidney Injury” ^12^. This mandated the installation of a new detection algorithm in each NHS hospital, so that potential AKI incidents could be flagged to treating clinicians. The ‘Think Kidneys’ NHS England National programme has provided best practice examples of how AKI alerts may be clinically deployed ^13^. However, simply alerting a clinician to the presence of a possible AKI incident may be insufficient to improve outcomes ^14^. A much richer clinical dataset is required to help clinicians prioritise, diagnose and manage patients. The UK’s National Institute for Health and Care Excellence (NICE) guidelines on AKI management suggest that patients with more severe AKI might benefit from care delivery by suitably expert clinicians, for example as part of a ‘rapid referral’ nephrology service ^11^.

The Royal Free Hospital (RFH) will implement existing standards for best practice through deployment of a digitally-enabled care pathway as a core service to hospital inpatients. A key component of this is a mobile software application (Streams-AKI). This application will identify potential new cases of AKI by applying the NHS AKI algorithm to a live stream of serum creatinine data, providing real-time monitoring of kidney function. The application will provide real-time alerts of potential AKI cases, alongside other critical clinical data, to a clinical response team comprising nephrologists and critical care nurses. This response team will assess the data provided by the application, prioritise cases and then deliver investigations and therapies according to current best practice guidelines ^11^. 

We propose a service evaluation of the introduction of the digitally-enabled care pathway with respect to processes of care, patient outcomes, qualitative feedback from patients and staff, and NHS costs.

## Aims and objectives

This service evaluation aims to assess the benefits of the implementation of the digitally-enabled AKI pathway from January 2017, with respect to clinical outcome, renal recovery, processes of care, and NHS costs. The evaluation will also assess the experience of service users (RFH in-patients who have been treated by the pathway), members of the clinical response team and the wider clinical community of RFH.

## Methods

### Service evaluation setting

The Streams-AKI app and clinical response team are deployed at a single hospital site within the Royal Free London Foundation Trust (RFLFT): the RFH, an 800-bed teaching hospital which also provides diverse specialist and tertiary services, including a dialysis unit and 34-bed intensive care unit with renal replacement therapy onsite. The outcomes of this evaluation will inform further development of the care pathway and its deployment in other RFLFT sites.

Following the implementation of the digitally-enabled care pathway, data from the RFH will be compared with data from the RFH prior to deployment in addition to pre-deployment and post-deployment data from a second hospital that is part of the RFLFT, Barnet General Hospital (BGH). BGH is a 450-bed district general hospital providing acute care (including onsite renal replacement therapy, a 21-bed ITU and on-site nephrology services), with similar arrangements for the care of AKI patients to that at the RFH prior to implementation of the digitally-enabled care pathway.

The RFH has onsite wireless internet networks available to clinicians, and was classified as a global digital exemplar (GDE) by NHS England in April 2016.

***The AKI care pathway prior to implementation*.** Serum creatinine, an indicator of kidney function, is currently measured in the hospital laboratory. The current AKI detection algorithm in the laboratory information management system (which predates the national algorithm) identifies potential AKI cases and presents a message for clinicians in both the hospital results system and electronic patient record. This message also flags the availability of clinical guidance and education via a link to a webpage displaying local guidelines ( www.londonaki.net). Prior to implementation, such results were normally batch reviewed by nonspecialists at the end of the day and may have been seen several hours after the results first become available. Clinicians may have opted to review results earlier, but this process relied upon repeated accessing of the results systems, as clinicians did not know when results were ready. Where blood tests suggested AKI, this may have been communicated by telephone to the clinical teams responsible for the patient by the biochemistry laboratory. However, this process was cumbersome and may have been unreliable.

Patients develop AKI in multiple wards and settings. Early management of AKI was overwhelmingly delivered by nonspecialist, ward-based teams with primary clinical responsibility for the patient. Specialist nephrology review of kidney function blood tests or of patients with AKI only occurred if requested by the patient’s responsible (or ‘home’) clinical team. This required the home team to assess kidney function results, assess the patient, decide to option a specialist review, contact the renal team via phone or pager systems and await a response. The renal team would then receive verbal referral information or would manually access results and other clinical data to prioritise the referral, managing information relating to multiple referrals with paper-based processes. The hospital’s critical care nursing team did not receive automated referrals and were entirely reliant on being contacted by pager systems when ward staff were concerned that a patient was deteriorating.

The RFLFT deployed clinical guidelines and had an active AKI education programme to support clinical teams, but local audit showed that performance in managing AKI varied, and did not always consistently meet national standards ^15^. Quality improvement in AKI care is a RFLFT organisational priority, and has driven the development of the care pathway and its proposed evaluation.

### The digitally-enabled care pathway comprising the Streams-AKI app and the AKI clinical response team

The digitally-enabled care pathway is the service whose implementation is being evaluated.

***The Streams-AKI application*.** Streams-AKI is a mobile application that is deployed on iPhone Operating System (iOS)-enabled smartphones. It processes routinely-collected demographic data (i.e. patient identifiers, location, responsible consultant, and responsible medical specialty), and also serum creatinine data in real time according to the nationally mandated NHS AKI algorithm, which grades patients’ AKI stage from 1 to 3. When the algorithm identifies a case of AKI, a patient-specific notification is delivered directly to the clinician user’s iOS device. In current clinical practice, clinicians must distinguish patients with clinically relevant changes in creatinine from those without, through review of current and historical blood tests, or elements of past medical history that indicate disease causality, complications or pre-existing risk. These routine data are therefore displayed in-app alongside the AKI alert to facilitate interpretation and clinical decision making. The Streams-AKI app is fully integrated with the existing RFH electronic health record system, and operates on Fast Healthcare Interoperability Resources (FHIR) interoperability standards. During implementation, it will be utilised alongside existing electronic health record software. Data security is ensured through the use of on-disk (AES256) and in-flight encryption (TLS v1.2) for all app data in compliance with NHS Digital information security guidelines. The app was first registered with the Medicines and Healthcare Products Regulatory Agency (MRHA) as a Class I, non-measuring, non-sterile medical device on 30/08/2016.

***The clinical response team*.** At the RFLFT, Streams-AKI will be installed on RFLFT-owned iOS devices. These will be held by members of a dedicated clinical response team, consisting of a clinical lead nephrologist, a duty consultant nephrologist, a specialty nephrology registrar, and a critical care outreach nurse. Following in-app review of alerts, all patients determined to be suffering from clinically-relevant AKI will receive a prompt bedside review form the nephrology team. The timeline to intervention after alerts has not been standardized, recognising that ‘real world’ clinical pressures and judgement may require prioritisation of actions within available resource constraints. We have, instead, specified immediate review for patients with life threatening results (e.g. hyperkalaemia), and that other, less urgent, cases are viewed within hours. The Streams app allows clinicians to view results and triage patients for early review based on perceived clinical urgency. The timeframe for review will therefore depend on clinical prioritisation and other (clinically determined) variable demands.

Members of the clinical response team will then administer a standardized care protocol. The critical care outreach nurse will also receive alerts on more severe (stage 2 and 3) cases and will respond to the most severely unwell cases according to clinical judgement of patient risk. All interventions and future care requirements will be communicated to responsible clinicians verbally and through a standard written proforma ( Supplementary Figure 1) entered into the patient record. Where necessary, the clinical response team will arrange a further review within 24 hours. The Streams-AKI app will further alert the team if the patient’s AKI stage subsequently worsens and will also alert after 48 hours if a patient is still suffering from AKI, as determined by the national AKI algorithm. The team will respond to such follow up alerts according to best practice and clinical judgement.

All clinicians using the app first receive training. This comprises:

- A detailed review of the Streams-AKI app
- Introduction to the devices being used to host the app: RFLFT-owned iPhones (Apple, Inc., Cupertino, Calif., USA)
- An introduction to the other members of the response team
- Review of the standardised digitally-enabled AKI care protocol.

**Figure 1.**
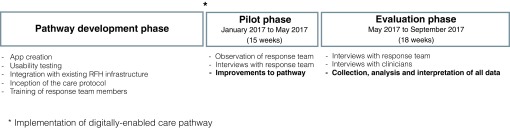
Evaluation phases. Phases of the evaluation are listed in order. A summary of the main aims of each phase is also listed. The digitally-enabled care pathway was implemented in January 2017.

Streams-AKI was deployed in January 2017. Following a pilot phase of 12 weeks to allow optimisation of the care pathway, an 18-week evaluation phase commenced, during which outcome data will be accrued (see Figure 1). For comparative analysis, data will be collected from the two hospital sites (RFH and BGH) at three time points:

- One year before deployment (January to August 2016)
- Immediately before deployment (September 2016 to January 2017)
- During deployment (January to August 2017)

An interim analysis is planned half way through the evaluation phase. Results from this may inform the duration of the service evaluation.

***Evaluation sample*.** The service implementation evaluation will include data from all inpatients triggering an AKI alert as defined by the national AKI detection algorithm who are aged 18 or over.

Utilising a daily data feed from the RFLFT chronic dialysis database, AKI alerts for patients receiving kidney dialysis will be identified by the application itself and removed. Alerts for inpatients on the Acute Kidney or Critical Care Units will also be removed. Patients on an end-of-life pathway at the time of the AKI alert will be excluded.

***Outcome measures*.** The evaluation of the pathway will employ a mixture of both quantitative and qualitative methods. The primary outcome will be recovery of renal function, which will be defined as a return to a creatinine level within 120% of the baseline (as defined by the National AKI algorithm) prior to discharge from hospital. Secondary outcomes will be categorised into four areas: processes of care, clinical outcomes, Trust-wide metrics, and NHS costs. Definitions of each outcome, and the sources of all data to be collected, are provided in Table 1– Table 4.

**Table 1.** Definitions and data sources for all process of care outcome measures.

Outcome measure	Definition	Source of data
Recognition of AKI	Time of documentation of recognition of AKI (digitally in-application or through written notes)	Electronic/Paper note review, data aggregated within the Streams data processor
Recognition of possible underlying cause	Time of documentation of possible cause(s) of AKI as: - Sepsis - Hypovolaemia - Obstruction - Nephrotoxins - Parenchymal disease	Electronic/Paper note review
Time to investigation	Time of documentation of: - Diagnostic imaging for obstruction - Diagnostic blood test or renal biopsy for parenchymal kidney disease	Electronic/Paper note review
Time to treatment	Time of documentation of: - Delivery of antibiotics for sepsis - Delivery of fluid for hypovolaemia - Relief of obstruction - Withdrawal of nephrotoxins - Definitive treatment for parenchymal kidney disease	Electronic/Paper note review
Time to specialist referral	Time of documented referral to: - Nephrology - Urology - High Dependency or Intensive Care Unit	Electronic/Paper note review
Time to specialist review	Time of documented review by: - Nephrology team - Urology team - High Dependency or Intensive Care Unit	Electronic/Paper note review

**Table 2.** Definitions and data sources for all clinical outcome measures. Health Level 7 (HL7) messages are used to transfer information between different healthcare IT systems.

Outcome measure	Definition	Source of data
Presence of AKI complications	Presence of: - Hyperkalaemia - Acidosis - Uraemia - Peripheral oedema - Pulmonary oedema During index admission	Electronic/Paper note review, HL7 data* aggregated within the Streams-AKI data processor
Recovery of renal function	Return to <120% index creatinine (as defined by NHS diagnostic algorithm) by the time of hospital discharge	HL7 data aggregated within the Streams-AKI data processor
Time to recovery of renal function	The time from AKI alert to recovery of renal function (<120% index creatinine).	HL7 data aggregated within the Streams-AKI data processor
Progression of AKI stage	Movement between AKI severity classes following AKI alert and prior to hospital discharge	HL7 data aggregated within the Streams-AKI data processor
Mortality	Death in 30 days following AKI alert	HL7 data aggregated within the Streams-AKI data processor
Length of stay	Time from AKI alert to hospital discharge	HL7 data aggregated within the Streams-AKI data processor
Admission to high acuity or specialist renal inpatient bed	Admission to: - Acute Kidney Unit (AKU) or other renal ward - High Dependency Unit (HDU) - Intensive Treatment Unit (ITU) During index admission	HL7 data aggregated within the Streams-AKI data processor
Length of stay in high acuity bed	Length of stay on: - AKU - HDU - ITU During index admission	HL7 data aggregated within the Streams-AKI data processor
Requirement for immediate temporary renal replacement therapy	Use of: - Haemofiltration - Haemodiafiltration - Haemodialysis - Peritoneal dialysis After AKI alert, but during index admission	HL7 data aggregated within the Streams-AKI data processor/ Trust submissions to the Health Episode Statistics- Admitted Patient Care database
Requirement long-term renal replacement therapy	Use of: - Haemofiltration - Haemodiafiltration - Haemodialysis - Peritoneal dialysis In 30 days following hospital discharge date	The Trust’s Nephrology Clinical Information Management System (VitalData) and Health Episode Statistics Admitted Patient Care database.
Readmission to hospital	Readmission to hospital in 30 days following index admission discharge date	HL7 data aggregated within the Streams-AKI data processor
Follow-up renal function	Last available creatinine obtained in outpatients or on discharge following another hospitalization between 90 and 365 days after index hospital discharge	HL7 data aggregated within the Streams-AKI data processor

**Table 3.** Definitions and data sources for Trust-wide outcome metrics.

Metric	Definition	Source of data
Referrals to critical care nursing team	Number of patients referred for review by critical care outreach nurses per 1000 bed days	Trust critical care nursing team logs
Unplanned admissions to ITU	Number of unplanned admission episodes in intensive care per 1000 bed days	Trust submissions to Intensive Care National Audit & Research Centre
Cardiac arrest rate	Number of cardiac arrests per 1000 bed days	Trust critical care nursing team logs

**Table 4.** Definitions and data sources for NHS cost outcome measures.

Cost Outcome	Definition	Source of data
Use of renal replacement therapy (resource use measured in days)	Use of: - Haemofiltration - Haemodiafiltration - Haemodialysis - Peritoneal dialysis	HL7 data aggregated within the Streams-AKI data processor/Trust submissions to the Health Episode Statistics- Admitted Patient Care database, and Payment by Results/local tariffs at the Trust
Length of stay on a regular inpatient ward	Length of stay outside: - AKU - HDU - ITU During index admission	HL7 data aggregated within the Streams-AKI data processor, and Payment by Results/local tariffs at the Trust
Length of stay in high acuity bed	Length of stay inside: - AKU - HDU - ITU During index admission	HL7 data aggregated within the Streams-AKI data processor, and Payment by Results/local tariffs at the Trust
Readmission to hospital	Readmission to hospital in 30 days following index admission discharge date	HL7 data aggregated within the Streams-AKI data processor, and Payment by Results/local tariffs at the Trust
Staffing costs	An estimate of staffing cost associated with the delivery of usual care vs. the *Streams-AKI* care pathway	Staff observation exercise
Cost of investigations	An estimate of costs associated with interventions made and/or investigations ordered as a result of AKI	Staff observation exercise, HL7 data aggregated within the Streams-AKI data processor

***Qualitative evaluation*.** During the 12-week pilot phase, the clinical response team was observed by a member of the service evaluation team, allowing key issues relating to both the technological and clinical aspects of the enhanced care pathway (including resource use) to be recorded. Semi-structured interviews were carried out with a selection of response team members (including nephrology consultants and specialty registrars, and critical care outreach nurses). The interviews explored whether the clinical response team members found that the new care pathway and the Streams-AKI application helped them provide best-practice care for patients, which aspects of the digitally-enabled pathway worked well or where they could be improved, adverse experiences or consequences of app use, and any unexpected indirect beneficial or adverse effects. Observational work and interviews carried out during the pilot phase were used to drive iterative improvements in the digitally-enabled care pathway prior to the beginning of the evaluation phase.

At the conclusion of the evaluation phase, a selection of doctors and nurses will be invited to a second series of semi-structured interviews. We will specifically target those responsible for the care of those patients who have been reviewed by the AKI response team during the evaluation. Participants will be purposively sampled to include a mixture of grades of clinicians (House Officers; Senior House Officers; Registrars; Consultants) and nurses (Staff Nurse; Charge Nurse). These will explore strengths and weaknesses of the digitally-enabled care pathway; how these map to perceived deficiencies in AKI care; how the new pathway affected the quality and equity of patient care; and how they feel the service could be improved. Approximately 20 interviews will be carried out. The exact number of health care professionals interviewed will be determined by the need to achieve sufficient diversity in elicited accounts for the questions to fully address all major themes, with no new issues arising at further interviews.

Figure 1 outlines the timeframes for each phase of the service evaluation, as described above.

### Statistical analysis plan and power calculation

We will use interrupted time series segmented regression analysis to examine the effect of the new AKI service on the weekly patient recovery rate for acute kidney injury within 30 days; recovery is defined as a return to creatinine level within 120% of baseline level. The primary dependent variable (recovery of renal function) will be modelled using a generalised linear model assuming a binomial distribution and using a logit link. This modelling approach will ensure that predicted values yielded from the model cannot fall outside the valid 0–100% range. We will also allow for autocorrelation in the model, which can be an issue with time series data. Using this approach, it will be possible to test for a change in level and/or regression slope following the implementation of the intervention.

Pathological and clinical endpoints will also be compared to those from a partner hospital (BGH) not deploying the digitally-enabled care pathway over the same time period, and from the pre-implementation periods as specified above. BGH has been included as a comparison site to allow us to demonstrate that impacts attributed to the service being evaluated are not due to systemic changes in process locally or nationally (e.g. relating to the National awareness campaign, ‘ *Think Kidneys*’). Baseline care pathways are similar at BGH and RFH.

It is anticipated that the care pathway will be subsequently deployed in BGH, with such deployment informed by the results of this service evaluation. Although comparison with the BGH comparator site should negate any seasonal effects, this is based on the assumption that the effect of time is the same in both intervention and comparator sites. The inclusion of the second pre-intervention period (i.e. immediately preceding the intervention period) will allow us to assess whether this is a fair assumption.

For the economic analysis, unit costs for each of the components will be obtained from two sources. First, we will use tariffs from the NHS National Tariff Payment System. Second, we will use local tariffs at RFLFT sites. Costs will be calculated using both sets of unit costs. We will multiply resource use by unit costs for each patient/cost component and sum across patients to calculate total costs per patient. The output will be a patient-level dataset of total costs per patient before and after the introduction of the digitally-enabled care pathway. Any attributable cost savings will be balanced against the additional costs of the alerting system and care pathway. This will include estimates of the use of clinician time; an activity observation exercise will be carried out with response team members during the evaluation period. A sensitivity analysis will be completed to assess the cost of care delivered to patients with AKI alerts that were discarded at clinician triage.

At the point of writing this service evaluation protocol, there are no published sample size calculations available for determining the number of timepoints needed for a well powered service evaluation using an interrupted time series design. In line with best practice in service evaluation, we therefore utilised simulations implementing the SIMSAM command in Stata (v14) (StataCorp LLC, College Station, Texas, USA) to establish the sample size needed. We simulated data containing weekly referral rates for four years prior to intervention, where the intervention will occur at 208 weeks. The average baseline recovery rate was assumed to be 0.51 (SD 0.08) which was determined using one year of pre-intervention data from the Royal Free Hospital, the site where the intervention will be implemented. One hundred observations (patients) or more per timepoint are encouraged ^16^, which is a viable assumption based on historical data. A normally distributed random variable with mean of zero and standard deviation of 0.08 was generated to simulate the variation in recovery rate. The pre-intervention regression slope and the change in the effect of the intervention over time following the intervention were both assumed to have an odds ratio of one. The recovery rate was generated as a function of these effects, the average baseline referral rate and the random variable.

The number of timepoints (measured on a weekly basis) needed to detect an odds ratio of 1.15 for the intervention effect with 90% power assuming a significance level of 5%, determined by simulation, was 11 in total. This number of post-intervention timepoints increased to 32 if the size of effect to be detected has an odds ratio of 1.1, i.e. a 10% increase in the odds of recovery. The number of timepoints needed to detect an odds ratio of 1.1 for the intervention effect with 80% power assuming a significance level of 5%, determined by simulation, was 20 in total. In further analyses, the segmented regression model will be extended to include a second hospital site for comparison of the change in level or change in slope post intervention. This will be Barnet Hospital, which will not receive the intervention.

All other quantitative data collected (see Table 1) will be analysed using Stata. Data will be screened for normality and homogeneity of variance prior to analysis.

A series of interim analyses will be performed midway through the evaluation phase. These will include inter- and intra-operator analyses of variance for transcription of process of care data from patient notes, and initial modelling of primary outcomes. These analyses will be carried out by staff from University College London and St George’s University of London.

***Analysis of qualitative data*.** The semi-structured interviews will be digitally recorded and transcribed verbatim. We will analyse the data from a realist viewpoint ^17^, but our model will be revised to fit emerging interpretations of the data. We will apply a framework approach ^18^, whereby the initial transcripts will undergo scrutiny by one member of the service evaluation team (AC) in order to gain familiarity with the data and to identify key themes. These will be discussed and further scrutinised with another service evaluation team member (RR), and with emerging interpretations or questions will be shared and critically explored with the with the entire service evaluation team. Transcripts will be analysed in a systematic manner by applying the coding framework and by rearranging the data according to the thematic content. Generalisations that represent the total set and that address each of the objectives will be developed. During analysis, we will maintain a constant vigilance for deviant cases that may question the emerging thematic and conceptual relations.

***Steering committee*.** A steering committee for this service evaluation has been convened at RFLFT. This committee includes an independent chair with no relationship to the project, a patient member and a nephrologist from a different NHS Trust. This committee will review the results of the interim analyses discussed above.

### Ethics, information and clinical governance

RFLFT uses Streams as part of its provision of care for patients. To support this service, DeepMind Health processes Patient Identifiable Data. This is in line with the governance arrangements for all other clinical software applications, and this arrangement forms part of an information processing agreement with the Trust, which has been published on the DeepMind website. The digitally-enabled care pathway will be a new standard service at RFH, and under NHS guidance there are therefore no consent requirements for patients for the processing of their personally identifiable data for direct patient care functions.

A determination by the Office of the Information Commissioner (ICO) was published in July 2017. This primarily focused on the clinical safety testing phase of Streams-AKI that occurred between app development (using only synthetic data) and the full clinical implementation that is being evaluated. The ICO ruled that the processing of data for the clinical safety testing of the application did not amount to processing for direct care purposes and so did not fully comply with data protection law. Concerns were also raised that not enough was done to ensure that patients and the public were aware of the project prior to data processing. RFLFT undertook such clinical safety testing in the interests of patient safety, but has accepted the determination by the ICO and is in the process of completing her specified undertakings. The Trust statement on this determination is available on their website.

When undertaking the service evaluation, data will be transferred from RFLFT to the Department of Applied Research, University College London for analysis. Prior to transfer and analysis, all data will be de-identified, meaning that no consent will be required from patients for this purpose.

Plans for the evaluation of the digitally-enabled care pathway have been independently reviewed by the University College London Joint Research Office. They directed that this project falls under the remit of service evaluation, as per “ *Defining Research*” guidance from the NHS Health Research Authority. As such, the service evaluation has been registered locally with the RFLFT Audit Lead and Medical Director. The service evaluation has the approval of the RFLFT Executive, RFLFT Board and Sub-Board Patient Safety Committee (including patient governor representatives and a non-Executive Chair and RFLFT Board Member). The Patient Safety Committee and RFLFT Board will receive reports on the results of the service evaluation. The service evaluation has also been presented to the UK’s National Institute for Health Research (NIHR) North Thames Collaboration for Leadership in Applied Health Research and Care (CLAHRC) Patient and Public Involvement Panel. The service evaluation has the full support of the Royal Free Kidney Patients Association.

It is theoretically possible that implementation of the digitally-enabled care pathway could have unforeseen adverse consequences. These will be sought through clinical feedback at weekly Trust implementation meetings and monthly patient safety programme operational group meetings. Additionally, during the service evaluation, broad metrics of care quality and safety will be monitored by the Trust, and these will be reviewed by the independent steering committee at RFLFT for this evaluation (described above).

### Dissemination of findings

Results will be presented to the renal, acute medicine and critical care departments, and to the RFLFT Patient Safety Committee and RFLFT Board. The findings will inform subsequent developments of the care pathway and will inform the RFLFT strategy for detecting and managing AKI. Data will also be presented on the Trust website via our patient safety portal and presented in lay language. Our findings will be shared at a learning event of the UCL Partners AKI Quality Improvement Collaborative, which has 9 participant Trusts as well as a London AKI Network event and as a case study to Think Kidneys (the NHS England National AKI Programme). We will publish in relevant scientific journals, and present at conferences.

## Supplementary Data

**Supplementary Figure 1: the standardized Streams-AKI proforma.** The outcomes of patient review (including suggested interventions and future care requirements) are communicated to responsible clinicians via a standard written proforma that is entered into the patient record.
