# Supplementary material for: Service evaluation of the implementation of a digitally-enabled care pathway for the recognition and management of acute kidney injury
Source: F1000Res. 2017 Aug 7;6:1033. Originally published 2017 Jun 30. [Version 2] doi: 10.12688/f1000research.11637.2 (PMC5510018; doi:10.12688/f1000research.11637.2)
Supplement: Supplementary file 1 [file f1000research-6-13358-s0000.tgz › 48e5be1d-4ab5-4f56-b325-17fe6eb53a3c.pdf]

Royal Free London NHS
NHS Foundation Trust

affix patient sticker here

This patient has generated an AKI alert on the Streams application.
The AKI response team are here to support and advise.
Ongoing management remains the responsibility of the home team unless otherwise agreed.

We recommend the following actions to STOP-AKI:

Section	Action	
**Management of life threatening complications of AKI**	**Critically ill:** Call PARRT (2525) or ITU (1030)	☐
	**Hyperkalaemia or acidosis:** commence medical therapy as per guidelines	☐
	**Fluid overload:** Commence diuretics, nitrates/oxygen (if necessary), fluid restriction	☐
**Sepsis and hypoperfusion**	**Sepsis:** complete Sepsis 6 care bundle	☐
	**Has an infection causing AKI:** send cultures, commence or escalate antibiotics	☐
	**Hypovolaemic:** Start bolus fluid protocol. Give 500mls crystalloid and reassess, repeat as necessary. Escalate to senior review after 2 litres bolus therapy	☐
	Commence maintenance IV fluids	☐
**Toxicity**	**Drug cessation or adjustment required**	☐
**Obstruction**	**Obstruction is possible and patient needs same day diagnostic renal USS** Please call Matteo Rossi for bedside USS on 07443101848. If out of hours then discuss with radiology (1462). If obstruction present please contact urology registrar on 1487 or on x39536	☐
**Primary Renal Disease**	**Perform urine dipstick**	☐
	If urine dip clear: order 'AKI diagnostic set (basic)' on Cerner	☐
	If blood or protein present: order 'AKI diagnostic set (glomerular)' on Cerner	☐

General advice

If in doubt, contact the AKI registrar on 07950860822 (day) or 07950843257 (night)

For guidelines and education, visit londonaki.net or download the London AKI app:

- Take 4 hourly observations & ensure an escalation plan is in place
- Commence a fluid balance chart, measure weights daily and set a daily fluid balance target
- Daily bloods: use 'AKI follow up' order set on Cerner and follow up to renal recovery
- Avoid contrast if possible. Consider prophylaxis where contrast absolutely necessary
- Review all medications at discharge
- If renal function does not return to baseline at discharge, contact AKI registrar for advice

Section	Option	
**Follow up**	We will only see if contacted by you or re-alerted in Streams due to worsening AKI	☐
	We will schedule a further review	☐
	We will take over care of patient	☐

TIME SEEN:______:______ DATE: ______/_____/_____ SIGNED:____________________________

Grade: Registrar ☐ Consultant ☐ NAME:____________________________
