# Supplementary material for: Service evaluation of the implementation of a digitally-enabled care pathway for the recognition and management of acute kidney injury
Source: F1000Res. 2017 Aug 7;6:1033. Originally published 2017 Jun 30. [Version 2] doi: 10.12688/f1000research.11637.2 (PMC5510018; doi:10.12688/f1000research.11637.2)
Supplement: Peer review history

# Peer review: Referee response for version 2

**DOI:** 10.5256/f1000research.13358.r24826

**Published:** 2017-08-07

**Authors:** Mitchell H Rosner

**Affiliations:** 1 Division of Nephrology, University of Virginia, Charlottesville, VA, USA

I have read this submission. I believe that I have an appropriate level of expertise to confirm that it is of an acceptable scientific standard.

# Peer review: Referee response for version 1

**DOI:** 10.5256/f1000research.12569.r23970

**Published:** 2017-07-13

**Authors:** Christopher J. Kirwan

**Affiliations:** 1 Department of Renal Medicine and Transplantation; Department of Adult Critical Care, Royal London Hospital, Barts Health NHS Trust , London, UK; 2 Barts and The London School of Medicine and Dentistry, London, UK

This is the publication of a protocol describing a much needed intervention study regarding the detection and treatment of AKI. This project is clearly described and is already very advanced and close to the completion stage.

 This project relies on a new technology to rapidly process patient data through the NHS England national algorithm for the detection of AKI. This technology then distributes AKI alerts, via an ‘app’ to a dedicated specialist multi disciplinary clinical team who will then act according to national guidelines. The aim is to improve outcomes of AKI.

 This project tackles a complex medical problem yet I feel is appropriately designed to try and provide answers to the following question; Does the provision of real time processing and delivery of patient data regarding AKI to the dedicated MDT of clinicians, allow the implementation of an intervention (AKI care bundles) that improves outcome.

 The main difficulty will be demonstrating that outcome has improved, as this in itself is very hard to define as a primary measure, and if it has not, where in the process has this failed. However, I think the methodology and the inclusion of a number of secondary outcome definitions is sensible, useful and achievable in the context of the clinical problem. There is only really one other study that, unsuccessfully, tried to answer similar clinical questions (Wilson *et al*) so the authors have minimal literature to guide them yet there approach is sound. The analysis will incorporate a before and after side to the study at the intervention hospital but there is also a second site control which again adds strength to the protocol.

 This study cannot be replicated without access to the technology stream AKI app, however, should this trial show benefit, a wider distribution by the sponsor to a number of different hospital types for a multicentre study would seem logical.

 I would however ask the authors to consider on further outcome measure metric. The literature is clear that renal function measured by serum creatinine at discharge following significant illness can over estimate renal function (Prowle *et al *CJASN). This may confound the interpretation of success by a d/c creatinine being within 20% of baseline. A series of follow up creatinine measures at 3 and potentially 6 months would be a more robust measure of AKI treatment success as the long term effects of AKI on renal function is potentially the most important success of early AKI intervention.

 Finally, following recent media coverage in the UK in relation a court judgement regarding the sharing of patient data between the RFLFT and DeepMind, can the authors clarify for the benefit of all readers that this project remains legally and ethically sound.

 I very much look forward to the results of this important study.

I have read this submission. I believe that I have an appropriate level of expertise to confirm that it is of an acceptable scientific standard.

# Response

**Published:** 2017-08-03

**Authors:** Alistair Connell

**Affiliations:** UCL, UK

We thank Dr. Kirwan for his helpful comments, to which we are happy to be able to respond:

 

1. We agree that structured recording of follow-up creatinine at three and six months would be highly desirable. RFLFT does not currently have the infrastructure to implement long term hospital-based AKI follow-up for all patients with AKI exposed to the pathway under evaluation, nor do we have funding or resource for such work at this time. This may be a future development. However, we will present follow-up creatinine data from both intervention and comparator sites and time periods where these are readily available through existing clinical practice on the reviewer’s recommendation. Though hospital-based, structured AKI follow up clinics are not part of the current service improvement it is quite possible that more reliable detection and more rigorous early management will result in more reliable AKI follow up either through referral to renal clinic, non-renal secondary care clinics or through primary care follow up. We will attempt to study this effect.
2. We fully understand the reviewer raising this point, and agree that it requires clarification. The determination by the Office of the Information Commissioner (ICO) primarily focused on the clinical safety testing phase of Streams-AKI that occurred between app development (using only synthetic data) and the full clinical implementation that is being evaluated. The ICO ruled that the processing of data for the clinical safety testing of the application did not amount to processing for direct care purposes and so did not fully comply with data protection law. Concerns were also raised that not enough was done to ensure that patients and the public were aware of the project prior to data processing. RFLFT undertook such clinical safety testing in the interests of patient safety, but has accepted the determination by the ICO and is in the process of completing her specified undertakings. The Trust statement on this determination is available on their website: https://www.royalfree.nhs.uk/patients-visitors/how-we-use-patient-information/information-commissioners-office-ico-investigation-into-our-work-with-deepmind/

# Peer review: Referee response for version 1

**DOI:** 10.5256/f1000research.12569.r23972

**Published:** 2017-07-03

**Authors:** Mitchell H Rosner

**Affiliations:** 1 Division of Nephrology, University of Virginia, Charlottesville, VA, USA

The authors provide the rationale and study design for the implementation and evaluation of a digitally-enable care pathway for the recognition and management of acute kidney injury (AKI). The paper is well-written and the topic is timely given the impact of AKI on short and long-term outcome. This program has the potential to radically change how patients with the earliest signs of AKI are managed and the team should be applauded for these efforts. I had just a few minor queries:

1. It might be nice to show the actual protocol for intervention based upon the NICE guidelines
2. Is the timeline to intervention once the alert triggered standardized?
3. I am not sure I understand the rationale behind including Barnet General Hospital as compared to RFH- it would seem that the patient mix (cardiac surgery, etc) might be very different between these centers. Is there any methodology used to match causes of AKI and severity of illness?

Overall, this is an exciting study with potentially profound implications for care of patients with AKI.

I have read this submission. I believe that I have an appropriate level of expertise to confirm that it is of an acceptable scientific standard.

# Response

**Published:** 2017-08-03

**Authors:** Alistair Connell

**Affiliations:** UCL, UK

We thank Prof. Rosner for his insightful comments, to which we are happy to be able to respond:

 

1. We have appended the care protocol for the intervention as a supplementary figure.
2. The timeline to intervention after alerts has not been standardized, recognising that ‘real world’ clinical pressures and judgement may require prioritisation of actions within available resource constraints. We have, instead, specified immediate review for patients with life threatening results (e.g. hyperkalaemia), and that other, less urgent, cases are viewed within hours. The Streams app allows clinicians to view results and triage patients for early review based on perceived clinical urgency. The timeframe for review will therefore depend on clinical prioritisation and other (clinically determined) variable demands.
3. Barnet General Hospital has been included as a comparison site to allow us to demonstrate that impacts attributed to the service being evaluated are not due to systemic changes in process locally or nationally (e.g. relating to the English national awareness campaign, ‘Think Kidneys’). Resources do not allow us to implement the new care pathway simultaneously in Barnet and Hampstead sites, though we plan to implement the new service on the Barnet site as part of our staged service improvement plans. Learning from this evaluation will inform that process. We anticipate site-specific variation in outcomes relating to casemix, as Professor Rosner rightly points out. Baseline care pathways are, however, similar at Barnet and Hampstead sites: there is a legacy alert system in the results viewing platform, non-specialists undertake batch review of results, biochemistry staff phone out some AKI results, early care is administered by unsupervised non-specialist teams with discretionary escalation to expert input via bleep or phone referrals. We thus feel it is a useful site to compare process outcomes and time-series trends in both process and outcome measures. We will collect demographic and clinical data for all patients with AKI at both sites during the time periods stated, and will present descriptive comparison data at the point of publication.
